# Clinical and therapeutic features of Plummer-Vinson syndrome in a Tunisian population: a case series

**DOI:** 10.11604/pamj.2023.44.21.35809

**Published:** 2023-01-11

**Authors:** Lassaad Chtourou, Manel Moalla, Hela Gdoura, Hend Smaoui, Ons Khrouf, Roua Kallel, Leila Mnif, Ali Amouri, Mona Boudabbous, Nabil Tahri

**Affiliations:** 1 Department of Gastroenterology and Hepatology, Hedi Chaker University Hospital, Sfax 3089, Tunisia,; 2 Sfax Medical School, University of Sfax, Sfax 3029, Tunisia

**Keywords:** Plummer-Vinson syndrome, dysphagia, iron deficiency anemia, endoscopic dilatation

## Abstract

Plummer Vinson syndrome (PVS) is a rare entity and most publications are case or series of cases. Thus, we report a series from southern Tunisia. Our aim was to analyse the epidemiological and clinical characteristics, the therapeutic modalities as well as the evolution of this pathology. Thus we carried out a retrospective study from 2009 until 2019. For each patient with PVS, we collected the epidemiological, clinical, paraclinical data and therapeutic modalities. A total of 23 patients were enrolled with a median age of 49.52 years [18-82 years] and a clear female predominance (M/F=2/21). The median duration of dysphagia was 42 months [4-92 months]. Moderate microcytic hypochromic anemia was noted in 16 patients. The anemia was without obvious cause in 60.8% (n=14) of cases. The main endoscopic finding was a diaphragm in the cervical area. Treatment was based on iron supplementation followed by endoscopic dilatation with Savary dilators in 90.9% (n=20) and balloons for 9.1% of patients (n=2). Dysphagia recurred in 5 patients after a median of 26.6 months [2-60 months]. Three cases of PVS were complicated by esophageal squamous cell carcinoma. In conclusion, our series confirms that PVS affects mostly women. Anemia is frequently noted in these patients. Treatment is based on endoscopic dilatation which is often an easy and risk-free procedure and iron supplementation.

## Introduction

Plummer-Vinson syndrome (SPV) also known as Kelly-Paterson syndrome or sideropenic dysphagia is a rare pathology that was first described by Kelly and Patterson in 1919, then 3 years later by Plummer and Vinson [1-3]. This syndrome mainly affects women between 40 and 70 years old. Diagnosis is based on a triad of upper dysphagia progressively worsening to solids, iron-deficiency anemia, and a diaphragm on the upper esophagus also called esophageal “web” [1]. Over the years, a marked decline in prevalence was noted. However, this syndrome was considered from its discovery as a rare entity in Africa with a prevalence that did not exceed 1/100000 [4]. Treatment is based on esophageal dilatations and iron supplementation [5,6]. The aim of our work was to determine the epidemiological and clinical characteristics of PVS, to analyze the therapeutic and evolutionary modalities of this disease and to compare our results with those of the literature in order to identify the particularities of our population.

## Methods

**Study design and setting:** Tunisia is located in North Africa bordering Libya to the southeast, Algeria to the west, and the Mediterranean Sea to the north. It occupies an area of 163,610 square kilometres, of which 8,250 are water. Sfax is a city in Tunisia, located 270 km (170 mi) southeast of the capital Tunis. Sfax has a population of 955 421 (census 2014). The main industries are phosphate, olive and nul processing, fishing (largest fishing port in Tunisia) and international trade. We conducted a retrospective, descriptive and analytical study to determine the epidemiological, clinical and therapeutic characteristics of PVS in Sfax. The study was piloted over a period of 11 years (from January 2009 to December 2019).

**Study population:** twenty-three patients were included in our study. These patients were hospitalized in the Gastroenterology department of the Hedi Chaker University Hospital in Sfax. Were included patients who responded to the clinical triad: dysphagia, iron deficiency anemia and esophageal diaphragm at endoscopy and had received endoscopic treatment. Patients in which PVS diagnosis was uncertain and whose data were unavailable were excluded from the study.

**Data collection:** for each patient, we collected the epidemiological, clinical, paraclinical and therapeutic parameters. Variables included geographic origin, habits such as geophagy, tea consumption, smoking and diseases history. They included also associated signs like asthenia, weight loss and regurgitation and laboratory, endoscopic and radiological results. All these data were available in the patients´ files. Upper endoscopy was performed in all patients at diagnosis and completed after treatment. Staged esophageal biopsies were performed after endoscopic dilation. A barium meal was also performed for all patients. Other specialized examinations and analyzes have been performed in few patients depending on the context as part of the etiological assessment of anemia or the search for associated complications.

Endoscopic treatment was standardized in all patients and consisted of Savary dilators or balloons. The procedure was performed under general anesthesia in two steps. The first step consisted of placing the guide wire in the esophageal lumen under endoscopic and fluoroscopic guidance. The second step consisted in introducing the Savary dilators with increasing diameter ranging from 5 to 14 depending on the nature of the diaphragm or dilatation using balloons. At the end of the procedure, an endoscopic look was done to check for the absence of complications and to complete the digestive exploration.

**Laboratory tests:** blood count and ferritinemia were the two blood tests that we collected from patients´ files.

### Definitions

Anemia corresponded to a hemoglobin (Hb) level of less than 12 g/dl in women and 13 g/dl in men. It was considered hypochromic when the mean corpuscular hemoglobin concentration (MCHC) was less than 28 g/ dL and microcytic when the mean corpuscular volume (MCV) was less than 80 femtoliters. It was considered mild, moderate or severe when Hb was >10g/dl, between 8 and 10 and < 8 respectively. Anemia was considered sideropenic if ferritinemia was < 18 ng/ml.

**Statistical analysis:** the statistical study was carried out using the SPSS 23 software (significant p < 0.05). The Shapiro-Wilk test was used to verify the normality of the distribution of quantitative variables for a population < 50. Qualitative variables were compared by Chi-2 and Fisher test and quantitative variables by the student test. For bibliography, we used Medline (PubMed), Scopus, Google scholar and Springer databases in French and English with the keywords: Plummer-Vinson syndrome, dysphagia, sideropenic dysphagia, iron deficiency anemia, endoscopic dilation, esophageal dilatation.

**Ethical consideration:** our study did not require written consent since it was retrospective and anonymity and confidentiality of data have been respected when collecting information.

## Results

**General characteristics of the study population:** over a period of 11 years, 23 patients were collected. The median age of our patients was 49.5 years [18 to 82 years]. The frequency of the disease was significantly higher after the age of 20 years with a peak in frequency between the 4^th^ and the 6^th^ decade. They were divided into 2 men and 21 women. Geophagy, tea consumption and tobacco were reported by 7, 2 and 2 patients respectively.

**Figure 1:**
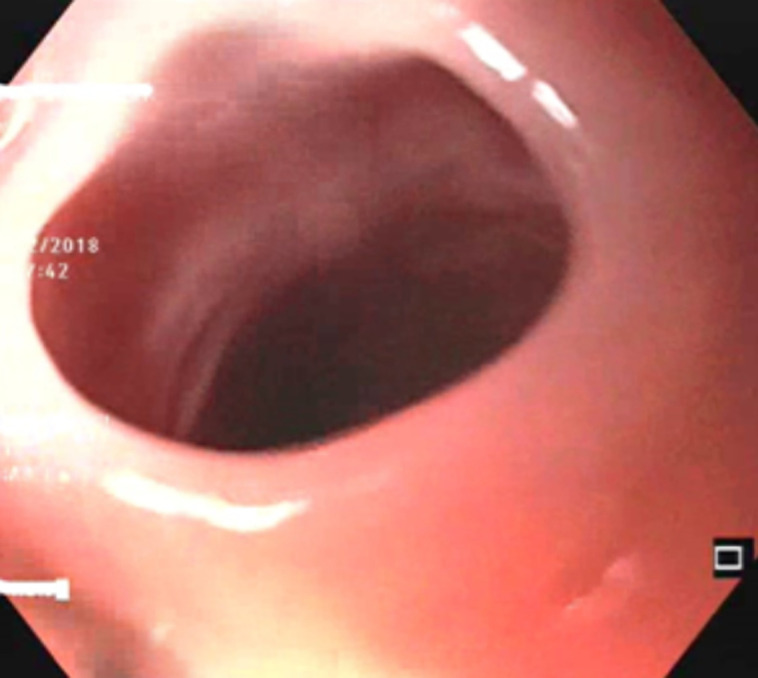
esophageal diaphragm in upper endoscopy

**Figure 2:**
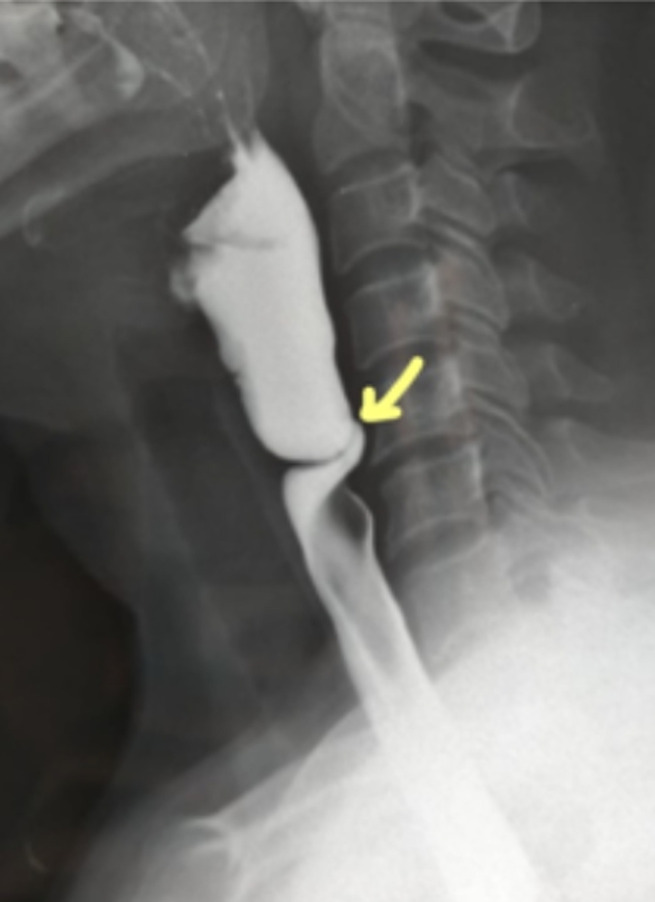
barium meal showing tight and short narrowing in the cervical esophagus

**Clinical presentation:** ten patients presented with a history of surgery. One patient had celiac disease and another one had hypothyroidism. Clinically, dysphagia was the primary and consistent functional sign described in all our patients. This dysphagia was progressive becoming severe in all cases. It was permanent in 17 patients (73.9%) and selective for solids in 20 patients (87%). The general condition assessment showed a PMS score = 1 in 5 patients. All patients had anemia, 7 of whom were already on iron supplementation. Hypoferritinemia was noted in 21 patients (91.3%). The two other patients whose ferritinemia was normal were already under iron treatment. The etiological investigation of iron deficiency revealed celiac disease in 2 patients and a gynecological origin in 1 patient. No etiology was found in 60.8% (n=14) of cases. Three patients had geophagia associated to another cause of anemia. The Table 1 summarizes the main findings. At initial upper gastrointestinal endoscopy, a diaphragm was present in the cervical esophagus in all patients (Figure 1). It was circumferential in 15 patients and semilunar in 8 patients. Barium meal showed cervical stenosis in all patients (Figure 2). Esophageal dilation upstream of the stenosis was noted in 5 patients (21.7% of patients). Barium meal results were consistent with upper endoscopy in terms of diaphragm seat in 100% (n=23) of cases and whether it was complete or not in 95% (n=22) of cases.

**Table 1:** epidemiological, clinical and biological characteristics of the study population

Characteristics	Data		Results
Epidemiological	Median age		49.52 years
	M/F		2/21
	Geographic origin (rural/ urbain)		56.5% / 43.5% (n=13/10)
Clinical	Dysphagia		100%
	Median duration of dysphagia		42 months
	Asthenia		43.5% (n=10)
	Weight loss		30.4% (n=7)
	Regurgitation		13% (n=3)
	BMI (mean)		20.86 ± 4.1 kg / m^2^
	Physical signs of sideropenia		39.1% (n=9)
Biological	Anemia	Moderate anemia	69.6% (n=16)
		Severe anemia	30.4% (n=7)
	Hypoferritinemia		91.3% (n=22)

Abbreviations: BMI: Body mass index; M: Male; F: Female

### Management and outcome

As part of the treatment, hygienic and dietary rules were recommended for all our patients, such as interruption of geophagy and tea consumption as well as smoking cessation. A diversified diet rich in animal proteins was also recommended. Iron supplementation was administered to all patients, orally in 21 patients (91.3%) and intravenously (initially) in 2 patients (8.7%), followed by oral relay. Endoscopic dilation was indicated and performed in 22 patients. It was unnecessary in 1 patient who was completely asymptomatic after initial endoscopy which caused the dilaceration of the diaphragm that was already loose. Endoscopic dilatation was performed using Savary´s dilators in 20 patients (90.9%) and balloons in 2 patients (9.1%). An average of 1.29 dilation sessions [1-4] was required for our patients. All dilation sessions were followed by a complete endoscopic check of the esophagus, stomach and duodenum with systematic biopsies of the stenosis. No immediate or distant complications were described in our patients.

In 16 patients (76.2%), the dilation associated to iron supplementation were sufficient for clinical improvement (disappearance of dysphagia) with a median follow-up of 38.6 months [2-96 months]. Dysphagia recurred in 5 patients after a median time period of 26.6 months [2-60 months]. A second dilatation session was effective for 3 patients while another patient with refractory anemia required 4 sessions of endoscopic dilatation (with intervals of 1 and 8 months respectively). We compared patients who required a single endoscopic dilation session versus those who required more than one session and only geographic origin was associated with disease recurrence (p = 0.031). In our series, PVS was complicated by esophageal squamous cell carcinoma in 3 patients being synchronous in 2 of them.

## Discussion

In our case series, we aimed to determine the epidemiological and clinical characteristics of PVS also known as sideropenic dysphagia or Kelly-Paterson syndrome, to analyze the therapeutic and evolutionary modalities of this disease and to compare our results with those of the literature in order to identify the particularities of our population. The main findings of our study were a median age of 49.52 years [18-82 years] and a clear female predominance (M/F=2/21). The most frequent revealing symptom was dysphagia present in all our patients with a median duration of 42 months [4-92 months]. Anemia was noted in all patients. Moderate microcytic hypochromic anemia was noted in 16 patients. The anemia was without obvious cause in 60.8% (n=14) of cases. The main endoscopic finding was a diaphragm in the cervical area. Treatment was based on iron supplementation followed by endoscopic dilatation with Savary dilators in 90.9% (n=20) and balloons for 9.1% of patients (n=2). Dysphagia recurred in 5 patients after a median of 26.6 months [2-60 months]. Three cases of PVS were complicated by esophageal squamous cell carcinoma. Two of them were synchronous to PVS diagnosis.

PVS was described mainly in Caucasian women from rural areas of northern Europe, exceptionally in men. The average age was between 40 and 70 years old. These data are consisting with our findings. It typically manifested as progressively worsening upper cervical dysphagia concerning solids in women with iron deficiency anemia [7]. This anemia may be missing in the series reported but the sideropenia was permanent. The diagnosis is based on a greyish complete or semilunar diaphragm at the entrance to the esophagus in upper endoscopy and a linear narrowing of the cervical esophagus in the area of C5-C6 in barium meal [1]. At endoscopy, the appearance is variable ranging from minor forms where the diaphragm can be destroyed by simply passing the endoscope such as the case of one of our patients, to extreme cases of severe stricture. As a result, several radiological aspects have been described according to the degree of esophageal stenosis ranging from simple angulation of the anterior esophageal membrane to regular tight short stenosis. In all cases, what matters is the location of the diaphragm on the esophagus.

From an epidemiological point of view, PVS is a rare disease but exact prevalence is not available. In fact, by referring to a few old prospective studies, the prevalence was variable depending on the region of study and did not exceed 1% in England [2,8]. Currently with the progress of medicine and the improvement of socioeconomic conditions, a marked decline in the prevalence has been noted. Thus, PVS has become extremely rare. However, since its discovery it was poorly reported in Africa although it is a continent known for malnutrition [7]. This can be explained by its low contribution to scientific publications as well as the limitation of diagnostic and therapeutic means, especially in sub-Saharan Africa. By referring to the few retrospective series reported in Africa, a Moroccan study including 135 patients, a Senegalese one and some Tunisian series [9,10], it was found that the epidemiological-clinical elements were similar to those described in Europe but has higher frequency of black women and relapse after endoscopic treatment.

Several therapeutic attitudes have been attempted since Plummer's first series. The management can be summed up in a medical aspect by the correction of iron deficiency anemia which could be sufficient for some patients. However, endoscopic treatment was often necessary using either Savary dilators or balloons under fluoroscopic control [11-13]. Endoscopic dilatation is at low risk of complications and give good results. In our series, this procedure was safe in 100% of cases. Surgical therapy is left for tight refractory strictures [14,15]. However, the persistence of the anemia may be in cause of the esophageal diaphragm recurrence leading to new dilation sessions, hence the benefit of making a careful etiological investigation of the anemia. The series reported in this publication joined data from the literature of European and African series from an epidemiological point of view with a female predominance and the average age of fifty. From the etiopathogenetic point of view, an association has been observed in some patients with autoimmune diseases such as celiac disease or hypothyroidism [16].

Our publication also focused on the association of malignant esophageal tumors apart from complications of PVS, hence the need of exploration by a preliminary barium meal before any endoscopic dilatation which will be useful either for the diagnosis as well as exploration of the esophagus upstream. Indeed, the occurrence of neoplasms, particularly of the hypopharynx and cervical esophagus, during PVS has been described in several studies. However, the mechanism of carcinogenesis remains poorly understood [17]. Monitoring for esophageal carcinoma is recommended but not yet consensual [18]. The limitations of our study is that the number of patients included is small. This may be explained by the rarity of this disease. Series with a bigger population study are needed to have generalizable applications in practice.

## Conclusion

In conclusion, our series confirms that PVS affects mostly women with sideropenic anemia. Treatment is based on endoscopic dilatation using Savary dilators or balloons which is often easy with low complication risk and iron supplementation. Screening for esophageal cancer is mandatory at the moment of diagnosis and after dilation.

### What is known about this topic

- Plummer Vinson syndrom is a rare entity affecting most commonly women with anemia;
- It is characterized by a diaphragm in the upper part of the esophagus;
- Its treatment is based on iron supplementation and endoscopic dilations.

### What this study adds

- Geographical origin is the only factor associated to the disease recurrence in our study;
- Esophageal cancer may be associated to PVS at the moment of diagnosis.
